# Case Report: Whole-Exome Sequencing of Hypothalamic Hamartoma From an Infant With Pallister-Hall Syndrome Revealed Novel *de novo* Mutation in the *GLI3*

**DOI:** 10.3389/fsurg.2021.734757

**Published:** 2021-09-22

**Authors:** Yue Yang, Fang Shen, Xie-Pan Jing, Nu Zhang, Shang-Yu Xu, Dan-Dong Li, Ling-Li Zhou, Guang-Hui Bai, Huang-Yi Fang, Zhong-Ding Zhang, Chen Pang, Jian Lin, Han-Song Sheng

**Affiliations:** ^1^Department of Neurosurgery, Second Affiliated Hospital of Wenzhou Medical University, Wenzhou, China; ^2^Department of Surgery, Northern Hospital Epping, Epping, VIC, Australia; ^3^Department of Neurosurgery, People's Hospital of Mongolian Autonomous Prefecture of Bayingolin, Korla Xinjiang, China; ^4^Department of Pathology, Second Affiliated Hospital of Wenzhou Medical University, Wenzhou, China; ^5^Department of Radiology, Second Affiliated Hospital of Wenzhou Medical University, Wenzhou, China; ^6^School of the 2nd Clinical Medical Sciences, Wenzhou Medical University, Wenzhou, China

**Keywords:** molecular profiling, whole-exome sequencing, Pallister-Hall syndrome, *GLI3*, hypothalamic hamartoma, surgical resection

## Abstract

**Background:** GLI-Kruppel family member 3 (GLI3), a zinc finger transcription factor of the sonic hedgehog pathway, is essential for organ development. Mutations in *GLI3* cause several congenital conditions, including Pallister-Hall syndrome (PHS), which is characterized by polydactyly and hypothalamic hamartoma. Most patients are diagnosed soon after birth, and surgical removal of hypothalamic hamartoma in the very young is rarely performed because of associated risks.

**Case presentation:** A 7-month-old boy with PHS features, including a suprasellar lesion, bifid epiglottis, tracheal diverticulum, laryngomalacia, left-handed polydactyly and syndactyly, and omental hernia was referred to our service. His suprasellar lesion was partially removed, and whole-exome sequencing was applied to the resected tumor, his peripheral blood, and blood from his parents. Histopathology confirmed the diagnosis of hypothalamic hamartoma, and molecular profiling revealed a likely pathogenic *de novo* variant, c.2331C>G (p. H777Q), in *GLI3*. Magnetic resonance imaging follow-up 1 year later showed some residual tumor, and the patient experienced normal development post operation.

**Conclusions:** We presented a case of PHS that carries a novel *GLI3* variant. Hypothalamic hamartoma showed a distinct genetic landscape from germline DNA. These data offer insights into the underlying etiology of hypothalamic hamartoma development in patients with PHS.

## Introduction

Pallister-Hall syndrome (PHS) was first described in 1978 following the observation of a series of congenital features in a group of patients who died during the neonatal period (1). PHS was later identified as an autosomal dominant condition with characteristic phenotypes including hypothalamic hamartoma (HH), polydactyly, bifid epiglottis, and imperforate anus (2). Through advances in molecular testing techniques, *Gli Kruppel Family Member 3* (*GLI3*) was identified as the causative gene of PHS in 1997 (3). The availability of MRI even in remote regions contributes to a better delineation of intracranial lesions in patients with PHS. Indeed, patients with PHS are known to have a wide variety of phenotypes include anal, genitourinary, and respiratory abnormalities (4, 5).

*GLI3* plays an essential role in regulating the development of multiple organ systems. Therefore, it is not surprising that different pathogenic *GLI3* variants lead to various developmental anomalies (6). Accordingly, personalized management is preferred for patients with different *GLI3* defects based on molecular studies. Major surgery has been performed to improve patient chance of survival by correcting defects such as imperforate anus. Moreover, given that patients with mild phenotypical abnormalities are more likely to survive into adulthood, cosmetic surgeries are often needed to improve their quality of life (7). HHs are associated with endocrine defects, seizures, and developmental delay in patients with PHS, and their surgical resection, if required, remains technically challenging, especially in the very young age group (8). In this study, we have successfully partially removed a HH in a 7-month-old boy diagnosed with PHS. Whole-exome sequencing (WES) of the tumor and blood samples of the patient revealed that he carried a likely pathogenic *de novo* variant in *GLI3*. Short-term clinical follow-up showed normal development in the patient. The reporting of this study conforms to CARE guidelines (9).

## Case Presentation

**Figure 1:**
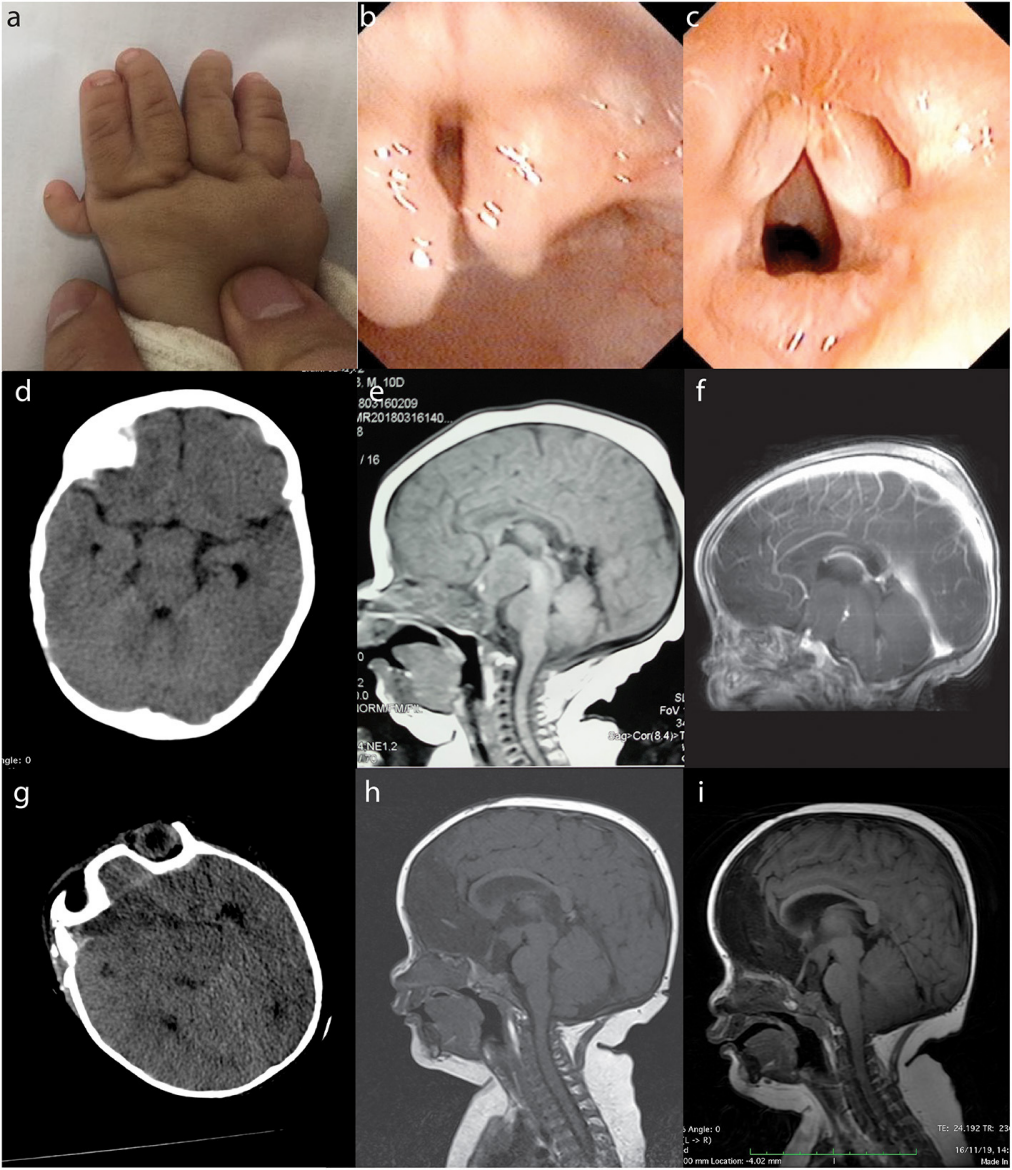
PHS in a 7-month-old boy. The patient presented with typical morphological features of PHS such as polydactyly and syndactyly (**a**: left hand), bifid epiglottis **(b)**, and laryngomalacia **(c)**. Preoperative CT (**d**: at 7 months of age), non-contrast MRI (**e**: 12 days postnatal), and contrast-enhanced MRI (**f**: 7 months of age) showed a suprasellar lesion. Postoperative CT **(g)** and MRI **(h)** revealed subtotal removal of the lesion, and follow-up MRI 1 year later showed no tumor regrowth **(i)**. PHS, Pallister–Hall syndrome; CT, computed tomography; MRI, magnetic resonance imaging.

A 7-month-old boy was referred to the Second Affiliated Hospital (also known as Yuying Children's Hospital) of Wenzhou Medical University after identification of an intracranial lesion by magnetic resonance imaging (MRI) scans that had been performed 12 days postnatally at a local hospital. He was born at full-term after an uneventful pregnancy by normal spontaneous vaginal delivery. At birth, he weighed 3.8 kg and was 50 cm in length. Physical examination on admission to our hospital revealed faltering growth (6.0 kg and 58 cm) [WHO criteria (10) at this age is 6.7–10.3, 8.3 kg (± 2 standard deviation, 50th percentile) and 64.8–73.5, 69.2 cm (± 2 standard deviation, 50th percentile)]. There were also dysmorphic features including left-hand polydactyly, syndactyly (Figure 1a) and a reducible omental hernia (1.5 × 1.5 cm). Flexible bronchoscopy showed bifid epiglottis, tracheal diverticulum, and laryngomalacia (Figures 1b,c). Neurological examination showed a normal muscle tone, strength, and reflexes. Endocrinology evaluations of pituitary, thyroid and cortisol hormones were within normal range. However, his parents reported that patient experienced frequent attacks of inappropriate laughter which suggested features of gelastic seizures. Unfortunately, electroencephalography was not available for such a small child to provide more objective evidence of seizure activities.

Computed tomography (CT) and MRI scans showed a suprasellar hypothalamic lesion suggestive of a hamartoma (Figures 1d–f). Surgery was performed because of parents' concerns over the faltering growth. The intracranial lesion was partially resected via transcallosal anterior interforniceal approach through the space between the internal carotid artery and optic nerve, and part of the tumor was left to avoid injuring the hypothalamus. The intraoperative diagnosis was hamartoma, and tumor samples were sent for histopathological and molecular testings. Postoperative course was uneventful, and repeated CT and MRI confirmed some residual tumor (Figures 1g,h). Patient was discharged 2 weeks later, and follow-up MRI 1 year later showed subtotal removal of the tumor (Figure 1i). Since the operation, he experienced normal growth without signs of gelastic seizures. At the clinical follow-up when he was 20-months-old, patient showed recovery of growth velocity (12 kg weight and 70 cm height). MRI at this time showed the residual tumor to be of a stable size and to be located in the tuber cinereum.

## Methods

### Immunohistochemistry

Staining was performed in the hospital pathology department using an automatic staining machine and commercially available hematoxylin and eosin (H&E) staining and immunohistochemistry agents. Briefly, tumor specimens were processed into 3- to 4-μm formalin-fixed paraffin-embedded section slides. For H&E staining, slides were first deparaffinized using xylene, rehydrated using ethanol, rinsed with distilled water, stained with H&E, and dehydrated. Slides were cover-slipped using Permount (Thermo Fisher Scientific, MA, USA).

For immunohistochemistry, antigens were first retrieved with either heat-induced epitope retrieval (62°C for 1 h) or citrate antigen retrieval buffer (95–100°C for 20 min). Then endogenous peroxidases were blocked with 1.5% hydrogen peroxide for 5 min. Slides were first incubated with primary antibodies against the following antigens including epidermal growth factor receptor (EGFR), glial fibrillary acidic protein (GFAP), epithelial membrane antigen (EMA), cytokeratins (CK), Sry-related HMg-Box gene 10 (SOX-10), S-100, P-glycoprotein (P-gp), cluster of differentiation 99 (CD 99), vimentin, neurofilament (NF) or synaptophysin (SYN) for 1–2 h per protocols. Then slides were incubated with horseradish peroxidase-linked secondary antibody (Thermo Fisher Scientific, MA, USA) for 30 min to 1 h at room temperature. All antibodies were ready to use without need of dilution. Diaminobenzidine tetrahydrochloride (Thermo Fisher Scientific, MA, USA) was sequentially added for 5 min then washed with phosphate-buffered saline for chromogenic detection. Slides were counterstained with hematoxylin before being mounted with Permount. All reagents were provided by Fuzhou Maixin Biotech Co., Ltd. (Fuzhou, China) and Beijing Zhongshan Golden Bridge Biotechnology Co. Ltd (Beijing, China).

### DNA Extraction and WES

DNA was isolated from tumor tissues, the patient's peripheral lymphocytes, and his parents' peripheral lymphocytes as described before (11). The quality of isolated DNA was assessed by using 1% agarose gel electrophoresis and Qubit^®^ DNA Assay Kit in Qubit^®^ 2.0 Fluorometer (Life Technologies, CA, USA).

The WES protocol was similar to the one we used previously (11). Briefly, DNA libraries were prepared using the Agilent SureSelect Human All Exon kit (Agilent Technologies, Santa Clara, CA, USA) following the manufacturer's recommendations, and index codes were applied to each sample. A total of 0.6 μg genomic DNA per sample was used as input material for DNA sample preparation. Library products were purified using the AMPure XP system (Beckman Coulter, Beverly, MA, USA) and quantified using a high-sensitivity DNA assay on the Agilent Bioanalyzer 2100 system (Agilent Technologies, USA). Clustering of the index-coded samples was performed on a cBot Cluster Generation System using the HiSeq PE Cluster Kit (Illumina, CA, USA) according to the manufacturer's instructions. After cluster generation, the DNA libraries were sequenced using the Illumina HiSeq platform, and 150 bp paired-end reads were generated.

### Variant Calling and Bioinformatics Analysis

FASTQ files of obtained exomes first underwent quality control to generate high-quality clean data. Valid sequencing data were then mapped to the reference human genome (UCSC hg19) by Burrows-Wheeler Aligner software to obtain initial mapping results in BAM format (12). SAMtools, Picard (http://broadinstitute.github.io/picard/), and Genome Analysis Toolkit (13) were used to sort BAM files and carry out duplicate marking, local realignment, and base quality recalibration to generate a final BAM file to compute the sequence coverage and depth. SAMtools mpileup (14) and bcftools performed variant calling and identification of single nucleotide polymorphisms (SNPs) and insertions and deletions (InDels). Somatic single nucleotide variants (SNVs) were detected by muTect (15), and somatic InDels by Strelka (16). Control-FREEC (17) was used to identify somatic copy number variation (CNV). The novelty of the mutation identified was verified by searching several publicly available databases, including ExAC, 1000 Genomes Project, and ESP6500. MutationTaster online software was used to evaluate the disease-causing potential of sequenced genetic alterations (18). Comprehensive visualization of gene mutations reported in the literature was performed by ProteinPaint, a web application for visualizing genetic lesions (19).

### Sanger Sequencing for Validation

The primers used for *GLI3* amplification were as follows: forward, 5'-TGAAACCCCAATCATGGACTC-3' and reverse, 5'-AGATGCATGGTCTGATGTAGAACTC-3'. PCR was conducted with 28 cycles of denaturation (94°C for 30 s), annealing (58°C for 30 s), and extension (72°C for 60 s). Reactions were performed in a Line-Gene 9600 Plus thermal cycler (BIOER, Hangzhou Bioer Technology Co., Ltd., Hangzhou, China). Reaction volumes were 20 μL that contained 1 μL DNA template, 1 μL each of forward and reverse primers, and 10 μL Premix Taq (Takara Bio Inc., Shiga, Japan) including deoxynucleotides and Tris-borate ethylenediaminetetraacetic acid as a buffer. Amplified DNA fragments were recovered from a low melting temperature agarose gel, purified with a Magnetic Beads Genomic DNA Extraction Kit (Enriching Biotechnology Ltd., Shanghai, China), and subjected to direct sequencing analysis using an automated ABI-3730 Sequencer (Applied Biosystems; Thermo Fisher Scientific, Inc., MA, USA).

## Results

### Histopathological Examination

**Figure 2:**
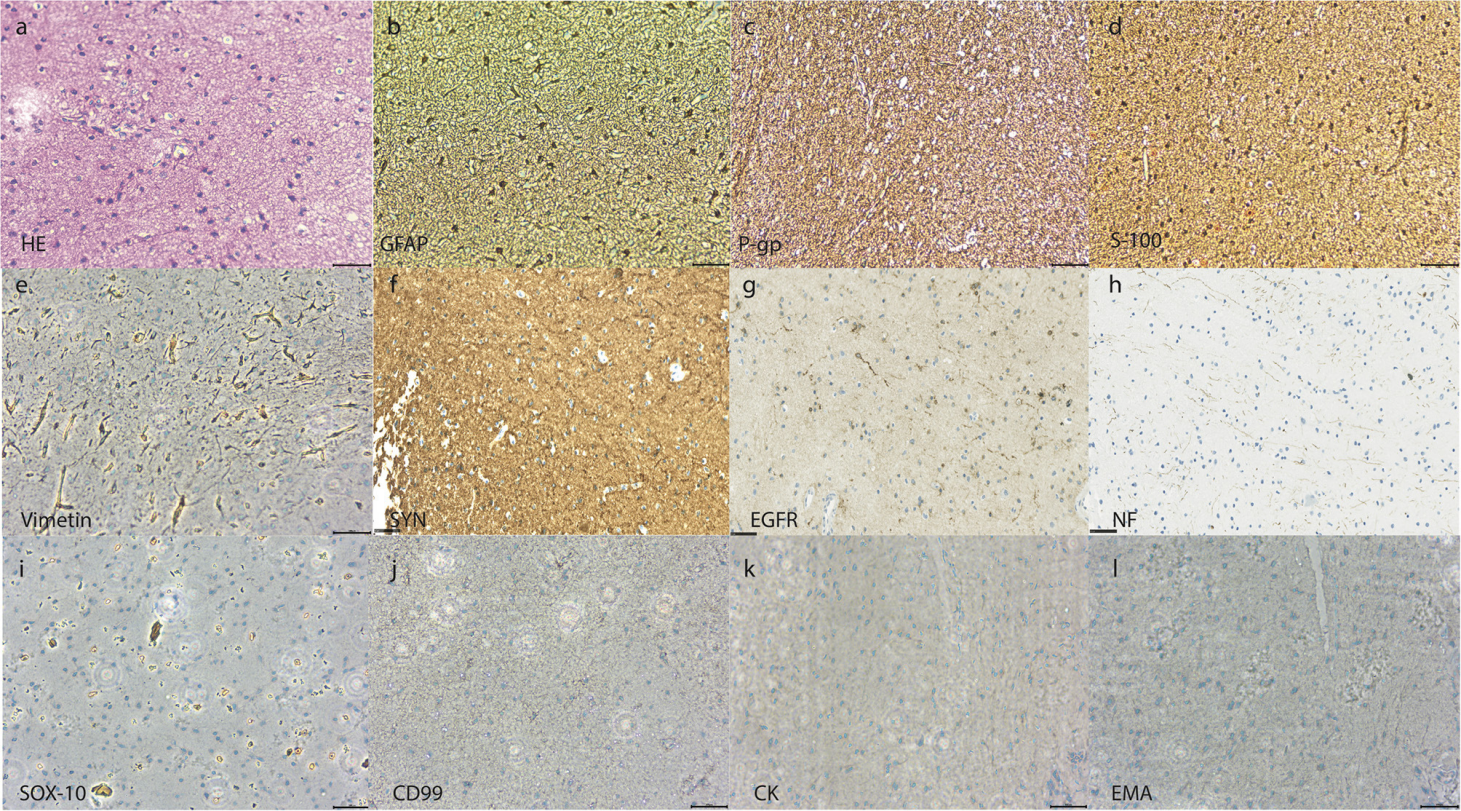
Pathological examination of the suprasellar hypothalamic lesion. H&E staining showing clusters of small neurons intermixed with glia and relatively sparse large neurons **(a)**. IHC (magnification ×400) showing positive staining for GFAP **(b)**, P-gp **(c)**, S-100 **(d)**, vimentin **(e)**, and synaptophysin **(f)**, and partial positive staining for EGFR **(g)**. Negative staining was seen for neurofilament **(h)**, SOX-10 **(i)**, CD99 **(j)**, CK **(k)**, and EMA **(l)** (Scale bar = 100 um). H&E, hematoxylin and eosin; IHC, immunohistochemistry; GFAP, glial fibrillary acidic protein; P-gp, P-glycoprotein; SYN, synaptophysin; EGFR, epidermal growth factor receptor; NF, neurofilament; SOX-10, Sry-related HMg-Box gene 10; CD 99, cluster of differentiation 99; CK, cytokeratins; EMA, epithelial membrane antigen.

H&E staining revealed clusters of small neurons intermixed with glia and relatively sparse large neurons. IHC analysis showed positive staining for GFAP, P-gp, S-100, vimentin, and SYN, and partial staining for EGFR. Staining for NF, SOX-10, CD99, CK, and EMA was negative. This staining pattern was compatible with a diagnosis of hamartoma with both neural and glial components (Figure 2) (20–22).

### Genetic Analysis

**Figure 3:**
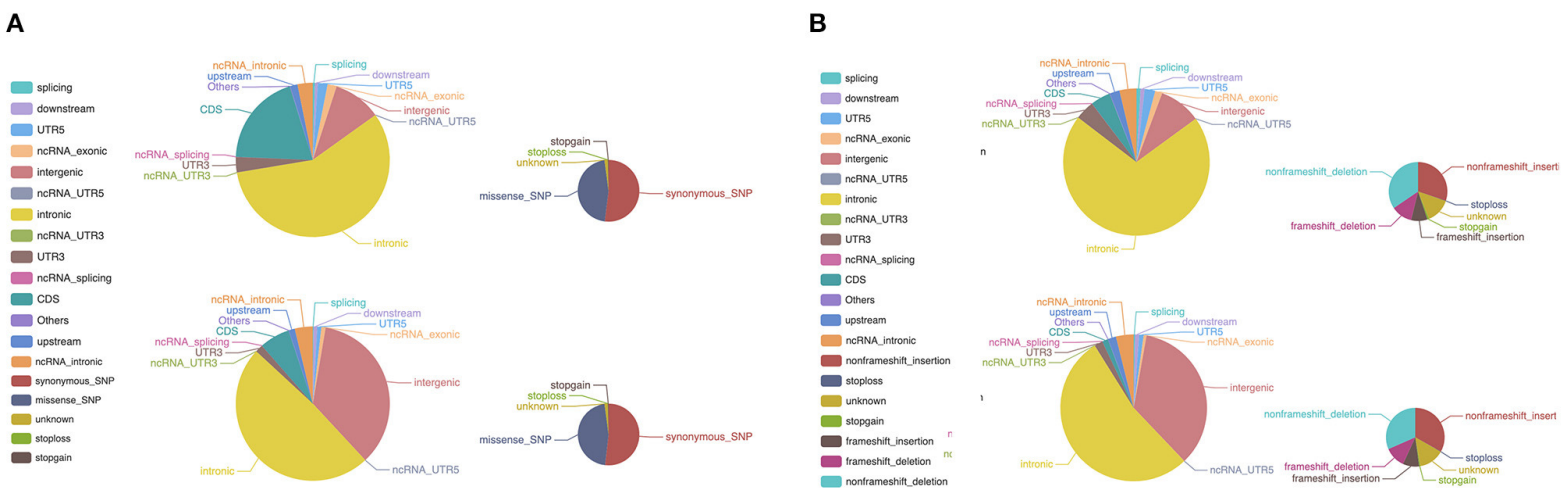
WES of the patient tumor sample and peripheral blood lymphocyte sample. **(A)** Differences in the distribution of SNPs between the tumor sample and peripheral blood lymphocyte sample. The larger pie charts represent the genome, and the smaller ones the CDS. **(B)** A similar pattern was observed in the distribution of InDels between the tumor sample and peripheral blood lymphocyte sample. The larger pie charts represent the genome, and the smaller ones the CDS. WES, whole-exome sequencing; SNP, single nucleotide polymorphism; CDS, coding sequence; InDels, insertions and deletions.

Cluster analysis showed similarity between peripheral blood lymphocytes samples from the patient and his father, while the tumor sample was more distinctive (data not shown). SNV analysis revealed that many SNPs in coding sequence (CDS) regions were common among the samples sequenced, with 45.39% of SNPs being shared by four samples, and 27.09% shared by three samples. A detailed comparison of SNP distributions in the genome and CDS regions showed differences between tumor samples and patient peripheral blood lymphocyte samples (Figure 3A). A similar trend was also observed in the InDel distribution in the genome and CDS (Figure 3B). These results indicate that the tumor gained further *de novo* mutations compared with germline cells. Somatic mutation analysis of the tumor sample revealed a total of 32 SNVs and six InDels. Among them, two SNVs (including missense SNV in POM121: c.A1456T:p.T486S) and one InDel (non-frameshift deletion of OLFM2: c.595_636del:p.199_212del) were identified in the CDS region. There was also a notable increase in CNV (*n* = 352) in the tumor sample compared with peripheral blood lymphocyte samples. These results suggest that the tumor sample had a different mutational burden compared with germline cells.

### Analysis of *GLI3* Variants

**Figure 4:**
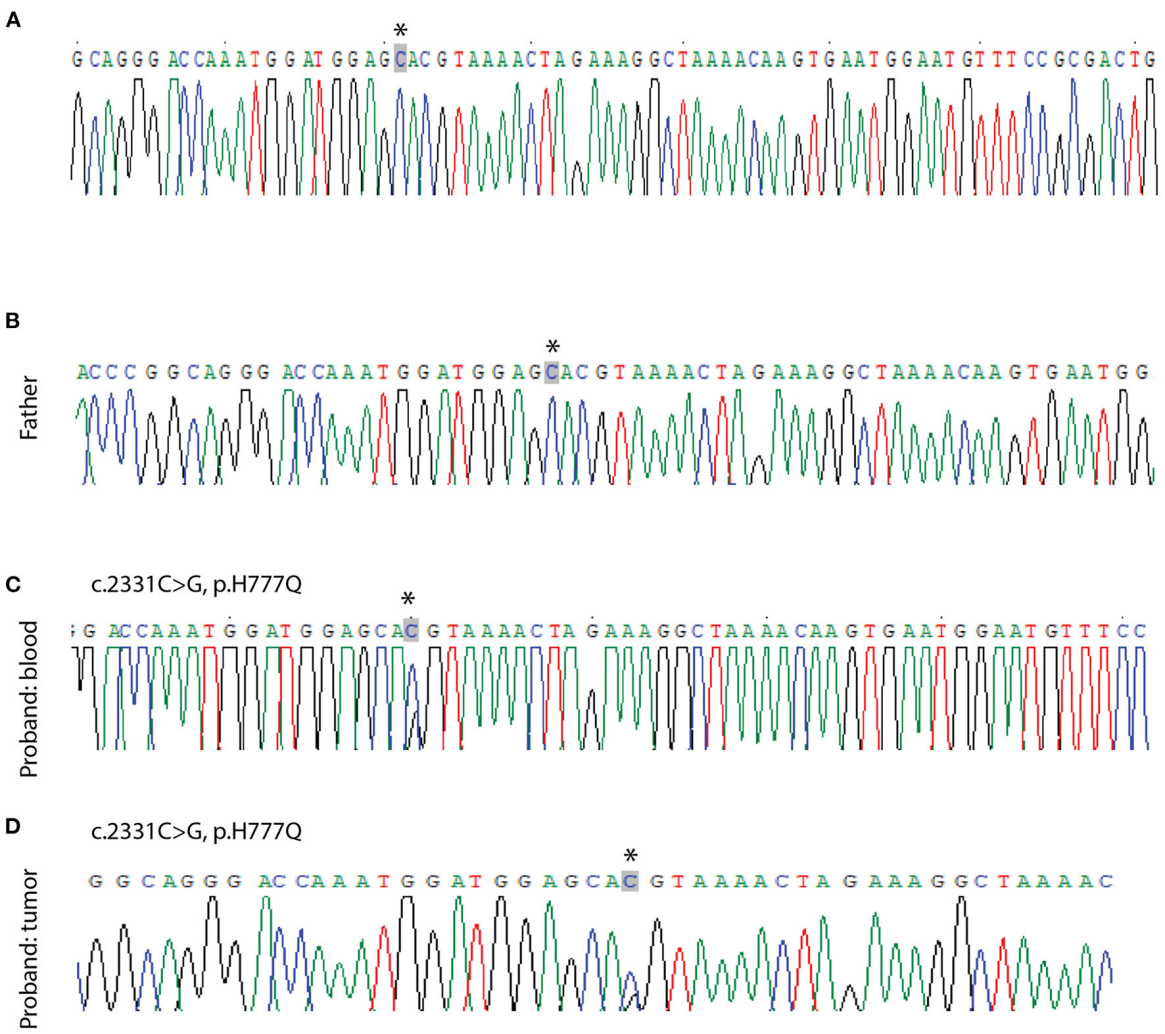
Sanger sequencing validation of point mutations in *GLI3*. No mutation is seen in the peripheral blood lymphocyte sample from the patient's mother **(A)** and father **(B)**. A c.2331C>G mutation is seen in the patient's blood sample **(C)** and tumor sample **(D)**. Arrows indicate the ivariant.

**Figure 5:**
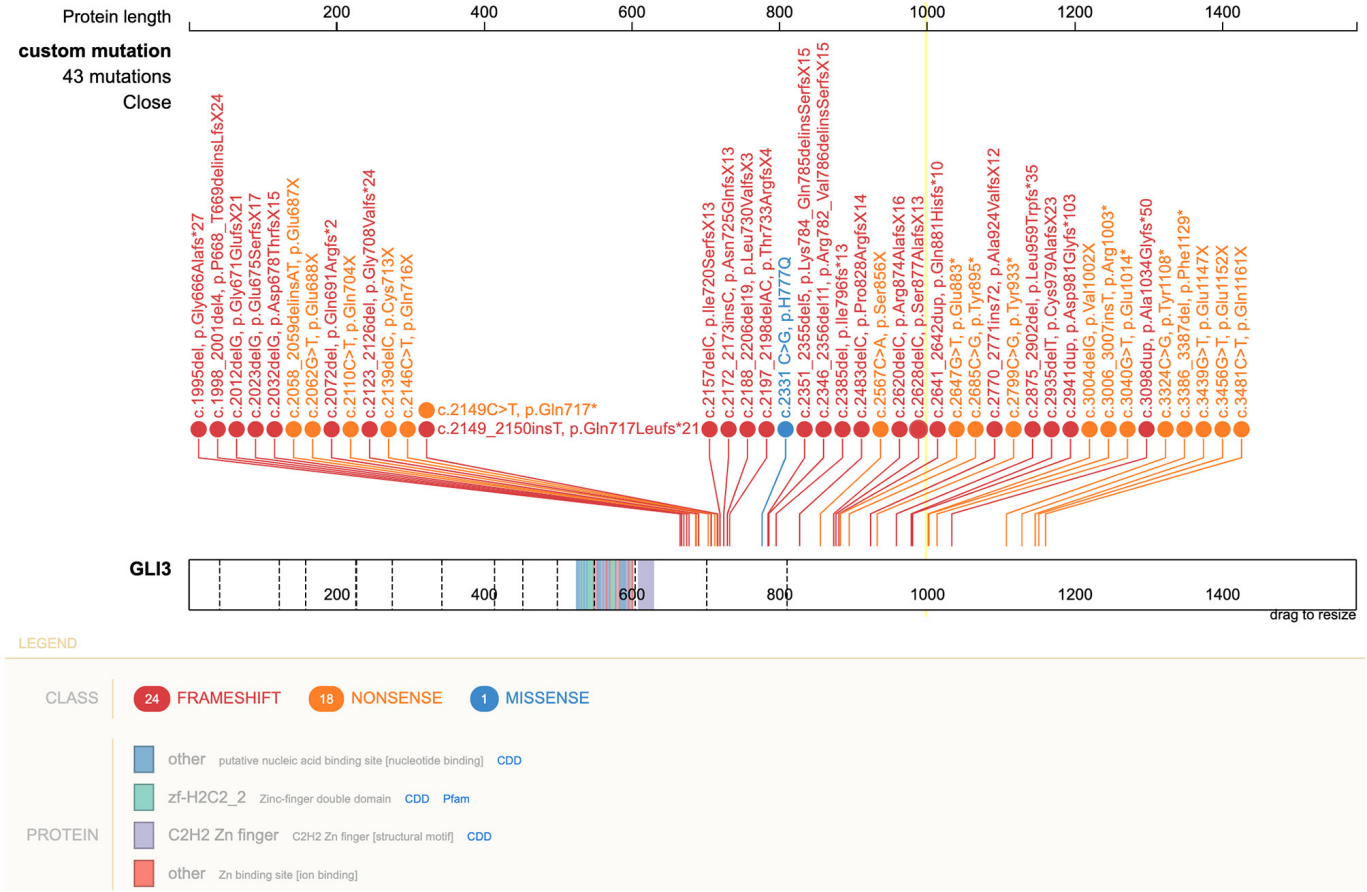
Schematic of *GLI3* and pathogenic variants associated with PHS reported to date. The pathogenic variant c.2331 C>G, leading to p.H777Q in *GLI3* is shown in blue. Right lower corner insert: GLI3 protein and location of variants. PHS, Pallister–Hall syndrome.

Following *GLI3* variants annotation, we observed variants c.C2993T (p.P998L) and c.A547G (p.T183A) in the germline cells of the patient and both parents, suggesting that they might be benign. The missense variant c.C2331G (p.H777Q) was predicted to be deleterious using MutationTaster and was only found in patient germline and tumor cells, suggesting a deleterious potential with *de novo* origin. This was further validated by Sanger sequencing (Figure 4). This variant was also absent from several public databases, including the ExAC, 1000 Genomes Project, and ESP6500. More than 40 pathogenic *GLI3* variants in CDS regions have been identified since 1993 in patients diagnosed with PHS (23). Literature review revealed this novel variant, c.C2331G (p.H777Q), was previously reported in a PHS patient with rare metopic suture fusion (24). These findings, together with our own, are shown in Figure 5.

## Discussion

PHS was originally described as a lethal condition in 1978 (25) with distinctive features including HH, postaxial or central polydactyly, anal atresia, and bifid epiglottis observed in the neonatal period (26, 27). It is now clear that PHS is not always lethal, and some patients survive into adulthoods (28). Clinical diagnosis of PHS would not be difficult when a constellation of phenotypical features as mentioned above is observed. Ideally, targeted genetic testing of the *GLI3* should be followed to confirm the clinical suspicion if resources are available (29). Nevertheless, early detection and personalized management such as endocrinal intervention for growth hormone deficits (30) improve patient outcomes. In sporadic HH, WES showed somatic mutations within genes comprising the sonic hedgehog (SHH) pathway (including *GLI3*) in up to 37% of cases (31). To our knowledge, this is the first report on WES of HH from cases of PHS which identified a novel *GLI3* variant both in tumor and germline DNA. Moreover, WES revealed additional *de novo* mutations in tumor DNA, indicating a different genetic landscape between tumor and germline DNA in our current case.

Despite mainly being of autosomal dominant inheritance, about 25% of PHS cases (32), include our patient, acquired through *de novo* mutations in *GLI3*. Defects in *GLI3* lead to dysregulation of the SHH signaling, which plays an essential role in multiple organ development. It was not until 2005 that the molecular mechanism underlying PHS was elucidated (33). This showed that most pathogenic mutations occur in the middle third of the *GLI3* gene (exons 13–15). To date, more than 40 pathogenic *GLI3* variants in patients with PHS have been reported (23). The majority of mutations are frameshift and nonsense mutations. In the present study, we identified the c.C2331G (p.H777Q) missense variant in our patient, which adds to the knowledge of pathogenic *GLI3* variants responsible for PHS phenotypes (34). This variant in exon 14 of *GLI3* was only previously reported in a PHS patient with a rare feature of metopic suture fusion (24). Interestingly, despite sharing the same variant, no metopic suture fusion was observed in our case on his CT scan at 7-months-old (data not shown).

Surgical management of HH at a very young age is challenging (35). Our report on the subtotal HH removal was performed in the youngest patient to date. The decision to operate was based on the following reasons: first and foremost, despite no obvious endocrinal/neurological deficits on examination, the patent's parents requested the operation because of the patient's slow growth rate and signs of gelastic seizures (30, 36). HH related epilepsy has been shown to start during the 1st year of life, typically as classic gelastic seizures (37) or less frequently as dacrystic seizures (38). Without surgical intervention, most gelastic seizures progress to tonic, myoclonic, or secondarily generalized seizures types (39). To minimize surgical-related trauma, only subtotal tumor removal was performed, which was confirmed by postoperative MRI. Follow-up MRI 1 year later showed no tumor regrowth, and the patient regained growth rate at the follow-ups. Nevertheless, Chibbaro et al. have described endoscopic resection of HH that lead to complete removal of the lesions and control of epileptic activity (40). This surgical advancement is important as it is paving the way toward minimal invasiveness management of those lesions, even in the very young.

Our study has several limitations. First, the surgical resection of HH might be arguable given its relatively benign biological behavior. Besides, surgical removal carries the risk of iatrogenic injury like any other intracranial procedure. Nevertheless, short-term follow-up showed our patient regained growth velocity without any complication. However, long-term follow-up is required to evaluate the benefits of early surgical intervention. Third, only two patients with PSH have ever been reported to carry the c.2331C>G (His777Glu) variant, and it is unclear how it alters the biochemical domains of GLI3 and subsequent signaling pathways. Further laboratory-based study is needed to elucidate this.

## Data Availability Statement

The datasets presented in this study can be found in online repositories. The names of the repository/repositories and accession number(s) can be found at: NCBI [accession: PRJNA743577].

## Ethics Statement

Written informed consent was obtained from the relevant individual(s), and/or minor(s)' legal guardian/next of kin, for the publication of any potentially identifiable images or data included in this article.

## Author Contributions

FS, YY, and X-PJ conceived and wrote the manuscript and performed sequencing studies. NZ and S-YX performed tissue biopsies. Z-DZ, S-YX, and D-DL performed data collection and statistical analysis. L-LZ performed pathological evaluations. G-HB performed radiological evaluations. H-YF, Z-DZ, and CP performed validation studies. JL and H-SS reviewed the manuscript. All authors contributed to the article and approved the submitted version.

## Funding

This study was supported by Zhejiang Province Science and Technology Program (Grant No. 2016C33213), Wenzhou Municipal Science and Technology Program (Grant No. Y20180665), Zhejiang Province Science and Technology Program (Grant No. 2021KY794), Wenzhou Municipal Science and Technology Bureau (Grant No. 2020Y0278), and Ningbo Natural Science Foundation (Grant No. 2018A610256).

## Conflict of Interest

The authors declare that the research was conducted in the absence of any commercial or financial relationships that could be construed as a potential conflict of interest.

## Publisher's Note

All claims expressed in this article are solely those of the authors and do not necessarily represent those of their affiliated organizations, or those of the publisher, the editors and the reviewers. Any product that may be evaluated in this article, or claim that may be made by its manufacturer, is not guaranteed or endorsed by the publisher.
